# On the challenges of predicting treatment response in Hodgkin’s Lymphoma using transcriptomic data

**DOI:** 10.1186/s12920-023-01508-9

**Published:** 2023-07-20

**Authors:** André Patrício, Rafael S. Costa, Rui Henriques

**Affiliations:** 1 INESC-ID and Instituto Superior Técnico, Universidade de Lisboa, Lisboa, Portugal; 2 LAQV-REQUIMTE, Department of Chemistry, NOVA School of Science and Technology, Universidade NOVA de Lisboa, 2829-516 Caparica, Portugal; 3 IDMEC, Instituto Superior Técnico, Universidade de Lisboa, Lisboa, Portugal

**Keywords:** Hodgkin’s lymphoma, Cancer, Machine learning, Gene expression, Data modeling, Discriminative patterns, Biclustering, Computational biology

## Abstract

**Background:**

Despite the advancements in multiagent chemotherapy in the past years, up to 10% of Hodgkin’s Lymphoma (HL) cases are refractory to treatment and, after remission, patients experience an elevated risk of death from all causes. These complications are dependent on the treatment and therefore an increase in the prognostic accuracy of HL can help improve these outcomes and control treatment-related toxicity. Due to the low incidence of this cancer, there is a lack of works comprehensively assessing the predictability of treatment response, especially by resorting to machine learning (ML) advances and high-throughput technologies.

**Methods:**

We present a methodology for predicting treatment response after two courses of Adriamycin, Bleomycin, Vinblastine and Dacarbazine (ABVD) chemotherapy, through the analysis of gene expression profiles using state-of-the-art ML algorithms. We work with expression levels of tumor samples of Classical Hodgkin’s Lymphoma patients, obtained through the NanoString’s nCounter platform. The presented approach combines dimensionality reduction procedures and hyperparameter optimization of various elected classifiers to retrieve reference predictability levels of refractory response to ABVD treatment using the regulatory profile of diagnostic tumor samples. In addition, we propose a data transformation procedure to map the original data space into a more discriminative one using biclustering, where features correspond to discriminative putative regulatory modules.

**Results:**

Through an ensemble of feature selection procedures, we identify a set of 14 genes highly representative of the result of an fuorodeoxyglucose Positron Emission Tomography (FDG-PET) after two courses of ABVD chemotherapy. The proposed methodology further presents an increased performance against reference levels, with the proposed space transformation yielding improvements in the majority of the tested predictive models (e.g. Decision Trees show an improvement of 20pp in both precision and recall).

**Conclusions:**

Taken together, the results reveal improvements for predicting treatment response in HL disease by resorting to sophisticated statistical and ML principles. This work further consolidates the current hypothesis on the structural difficulty of this prognostic task, showing that there is still a considerable gap to be bridged for these technologies to reach the necessary maturity for clinical practice.

## Background

Hodgkin’s Lymphoma (HL) is a type of blood cancer that originates in the lymphatic system, more precisely in lymphocytes, with the patient age peak of diagnostics occurring at the 20 s and 30 s. In 2018, HL represented 0.4% of all new tumors (79990 new cases) and 0.3% of all cancer deaths (26167 deaths) worldwide [1]. Survival of Hodgkin’s Lymphoma patients has significantly improved over the past years. Still, after initial remission, patients experience an elevated risk of death from all causes [2], such as cardiotoxicity diseases like myocardial infarction and congestive heart failure [3], and secondary cancers [4], diseases that are often treatment-related [5].

The current prognosis for HL is largely based on the International Prognostic Score (IPS) [6] which predicts for 5-year freedom from progression. Moccia et al. [7] concluded that this scoring does not identify with certainty low or high risk groups, and recommends the use of molecular markers and/or fluorodeoxyglucose Positron Emission Tomography (FDG-PET) scanning for this purpose. Despite the proven relevance of FDG-PET for HL prognostic, this medical exam is: (i) intrusive, with the need to inject a radioactive tracer; (ii) expensive, estimated at 1020 Eur per exam [8]; and (iii) impossible to perform in remote locations and ambulatory settings as it requires large machinery.

The transcriptional activity of tumor cells is a viable proxy candidate to assess regulatory response to treatment, thus being positioned as a possible alternative to the FDG-PET exam. Nevertheless, the role of differential gene expression in HL has not been exhaustively studied as it is a relatively rare cancer (2.86 cases per 100,000 persons annually [9]). Specific approaches, such as hierarchical clustering [10, 11], Cox regression [12] and sparse multinomial logistic regression [13] have been explored in other works, but some of the state-of-the-art machine learning (ML) approaches successfully applied to more common cancers have not yet been comprehensively employed.

In this context, this work aims to assess the predictability limits of an interim FDG-PET performed after two courses of Adriamycin, Bleomycin, Vinblastine and Dacarbazine (ABVD) chemotherapy treatment using gene expression profiling as a surrogate. To this end, a superior methodology combining ML advances with subspace-based data transaformations is proposed. Transcriptomic data of Hodgkin’s Lymphoma patients’ diagnostic tumor samples acquired by Luminari et al. [14] are used with the objective of better understanding the predictability of a patient’s response to a specific chemotherapy regimen. To this end, we resort to gene expression profiles obtained from Formalin Fixed Paraffin Embedded (FFPE) diagnostic tumor samples [15]. Although there are other datasets available with gene expression profiles of HL patients [16–19],^1^ these were not collected with the objective of studying the result of a FDG-PET after undergoing a chemotherapy regimen, hence not possessing the fundamental information for our analysis.

Our work advances the current status quo on this task described by Luminari et al. [14], which is grounded on a more traditional statistical analysis of the data, resorting to multivariate logistic analysis, filtering by Fold-Change (FC) and False Discovery Rate (FDR) values and multivariate logistic regression. In contrast, we conduct a thorough optimization and assessment of preprocessing and ML techniques to develop a predictor that can, at the moment of diagnosis, classify patients’ future interim PET after two courses of ABVD chemotherapy according to treatment response.

In addition to the end-to-end assessment of state-of-the-art predictors, our work proposes the use of biclustering principles to transform the original high-dimensional feature space into one consisting of features given by discriminative gene expression patterns, and shows that the new space yields relevant statistical properties. The gathered results show that this novel transformation yields statistically significant improvements on the predictive performance.

## Methods

**Fig. 1:**
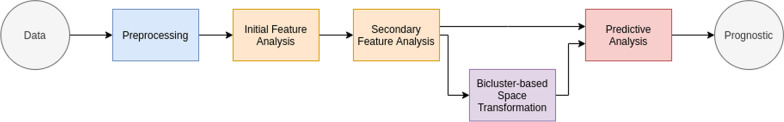
Schematic workflow of proposed solution for the treatment response prediction in HL disease

To tackle the introduced research problem, we propose the methodology presented in Fig. 1. This methodology starts with essential data preprocessing, followed by a feature analysis stage divided into two phases, resulting in a reduced data space conducive to the subsequent predictive analysis stage. Along the predictive analysis stage, we propose a bicluster-based space transformation that converts the gene-centric space to a pattern-centric one. State-of-the-art ML models can then be applied along the original or pattern-centric data space for the targeted prognostic ends. The methodology is implemented in Python (version 3.8.5) and the code is publicly available in GitHub.^2^

### Data

The proposed methodology is validated on the cohort study conducted by Luminari et al. [14], available at the National Center for Biotechnology Information Gene Expression Omnibus (GSE132348^3^). It consists of 106 samples of patients diagnosed with Classical Hodgkin’s Lymphoma. Each individual has associated the normalized expression levels of 765 different genes, obtained using the NanoString’s nCounter platform [15] over the RNA extracted from FFPE diagnostic tumor samples. Gender, age, stage of disease according to the Lugano classification [20], and Lymphocyte-to-Monocyte Ratio (LMR), LMR>2.1, are further provided. Finally, each record contains the result of an interim PET realized after two courses of ABVD chemotherapy (iPET2), which is classified as “positive” or “negative” according to the Deauville 5-point scale [21], with PET defined as positive when its ordinal value is greater or equal than 4. More information on the data collection process can be found in the original work [14]. The data is relatively imbalanced, with 84 (80%) iPET2 negatives, and 21 (20%) iPET2 positives.

### Data preprocessing

The mRNA counts were $\log _2$ transformed to better handle the variability of expression within and across genes. Samples with missing values were removed, resulting in a new distribution of 82 (79.6%) iPET2 negatives and 21 (20.4%) iPET2 positives. The variable *stage* was encoded as an ordinal variable, ranging from 1 (corresponding to“I A”) to 8 (corresponding to “IV B”), indicating that both a larger stage number and the B variant are worse prognostic factors. Since the dataset is considerably imbalanced, with a target’s distribution of around 80/20, balancing methods were assessed through a comparative analysis of their effects on predictive performance, with the combination of both oversampling and subsampling techniques being compared. Oversampling using Support Vector Machine Synthetic Minority Oversampling Technique (SVM-SMOTE)^4^ [23] yield the best results, hence being selected for the target experiments.

### Feature analysis

Given the high-dimensionality of the cohort data (770 transcriptomic features, 103 patients), dimensionality reduction was undertaken to aid the target predictive task. Following the practice suggested by Saeys et al. [24], and supported by previous empirical evidence [25–27], we pre-reduced the feature space using univariate filter methods Wilcoxon Rank Sum Test^5^ [29] and Mutual Information^6^ [31], and subsequently applied a more complex embedded method Support Vector Machine - Recursive Feature Elimination (SVM-RFE)$^{6}$ [32].

*Initial Feature Selection*. As most preprocessed features do not follow a normal distribution according to Shapiro-Wilk test [33], we resort to the non-parametric Wilcoxon Rank Sum Test [29] and Mutual Information (MI) [31] criteria. For each independent variable $y_j$ and the target response outcome $z$, the former Wilcoxon statistic tests whether the distributions $y_j|z=0$ and $y_j|z=1$ are equal. The latter MI statistic does not test a hypothesis, but a *p*-value is subsequently generated using a one-sided permutation test. As these approaches are univariate, not taking into account interactions between variables, a less strict than usual significance threshold of $0.1$ is considered to prevent the removal of potentially relevant genes.

*Secondary Feature Selection*. Grounded on empirical evidence [34], the embedded Support Vector Machine - Recursive Feature Elimination (SVM-RFE) [32], an adaption of RFE selection method that replaces an external ranking function with the magnitude of the weights of a Support Vector Machine, is subsequently employed.

### Predictive analysis

The literature on classification tasks in the oncotranscriptomics domain shows a relative predominance of specific machine learning (ML) models [35, 36]. Accordingly, Support Vector Machines (SVM) [37], k-Nearest Neighbors (KNN) [38] and Random Forest [39] are selected as classifier candidates. Given the consistently state-of-the-art performance of XGBoost [40] in other domains, we further disclose its predictability performance. Complementarily, Decision Trees [41] are further assessed as the learnt associations can reveal important insights about the genetic component of HL, and Naive Bayes [42] to offer a baseline stance on predictive accuracy.

In order to obtain the best classification possible, all the predictors are subjected to parameter optimization through the Tree Parzen Estimator algorithm [43], in addition to an optimized application of the aforementioned preprocessing techniques.

### Bicluster-based space transformation

The various biological mechanisms present in our bodies rarely originate due to a single gene’s expression values. Instead, the majority of gene regulation is done in a modular way, in which sets of genes interact with each other to enforce a certain mechanism. With this in mind, it is important to not reduce the analysis of transcriptomic data to individual genes but instead to study these values and its contributions in the context of putative regulatory modules. Furthermore, and in spite of the relevance of the introduced feature selection process, such process relies on univariate methods that do not take into consideration gene interactions and on an embedded method that show biases towards specific predictors [34].

In this context, we propose a novel transformation procedure in high-dimensional data spaces that can map individual gene expression features with loose discriminative power to discriminative pattern-centric features given by discriminative regulatory modules able to better represent complex interactions between multiple genes. Biclustering has been largely employed for the effective discovery of gene expression patterns [44–46], with pattern-based biclustering showing relevant performance indicators in diverse biological data contexts [47, 48]. Biclustering based on PAttern Mining Software (BicPAMS) [49] integrates dispersed state-of-the-art contributions on pattern-based biclustering, allowing for a high level of parametrization while performing efficient searches with guarantees of optimality, statistical significance and discriminative power [50, 51].

**Fig. 2:**
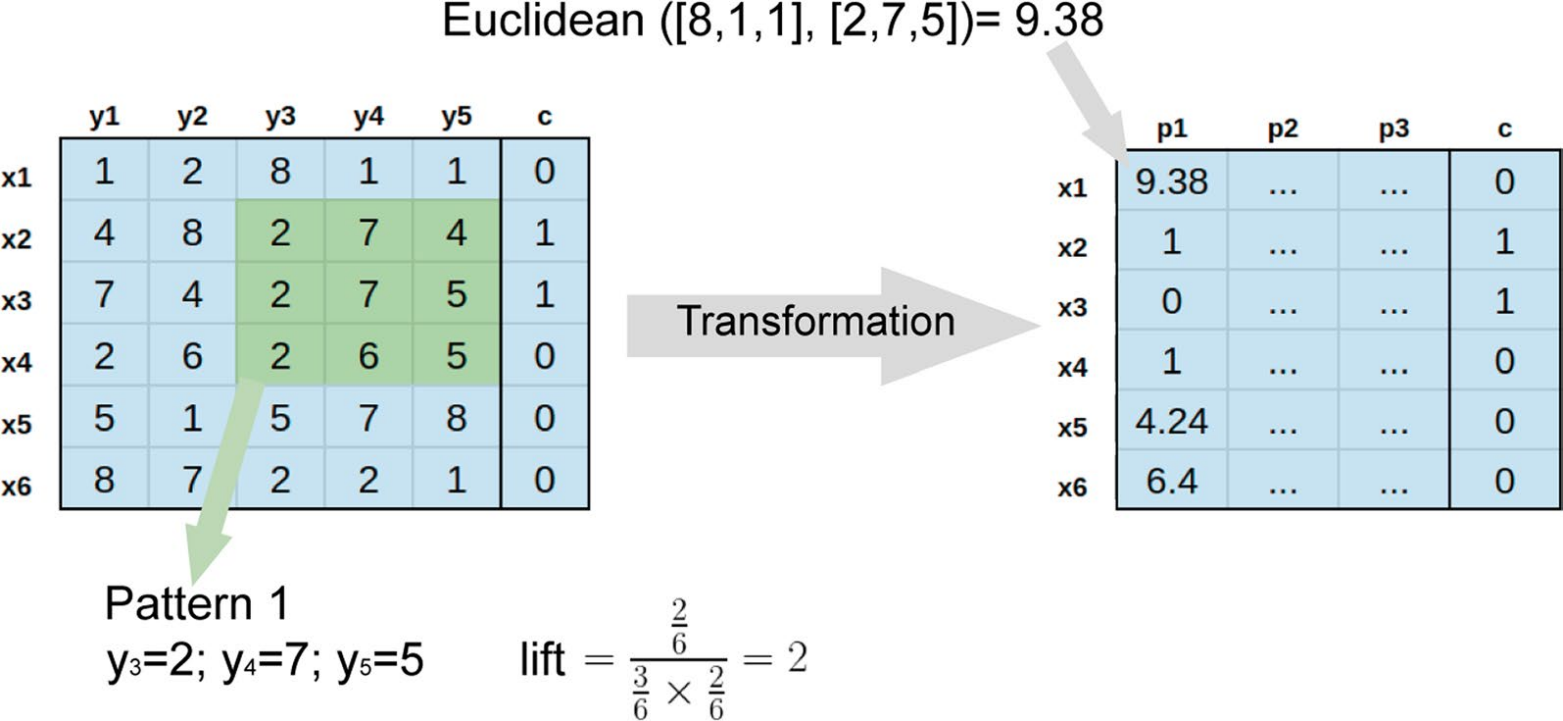
Example of space transformation using biclustering, showing a found bicluster with the corresponding pattern (*left*), and the resultant values on the transformed dataset using Euclidean distance (*right*)

In this context, BicPAMS is applied to find discriminative patterns on the training cohort data. Multiple parameterizations are tested in order to obtain the best space transformation for the classification task. The dataset is then transformed so that each feature corresponds to one gene expression pattern encompassing multiple genes. Each value of the transformed dataset will then represent the similarity between the gene expression expectations associated with a given pattern and the actual levels of gene expression observed for an individual. Figure 2 shows an example of the described process.

## Results

The proposed methodology is applied on the cohort study conducted by Luminari et. al [14] to assess the limits to the predictability of the quality of HL patient response to ABVD treatment. The gathered results are presented in two major steps: (i) a comparison of predictive levels from the optimized application of state-of-the-art ML models against the current reference levels; and (ii) an assessment of the improvement yield by the proposed pattern-centric data space transformation.

*Evaluation methodology*. Given the small population size (103 samples), a nested Cross-Validation (CV) schema is considered using 10 folds. Nested Cross-Validation is used to separate the data used for hyperparameterization from the data used for assessing the model’s performance [52]. Without this separation, the model would learn its parameters in data in which it would be tested, resulting in an overestimated predictive capability. In a nested CV, inside each CV loop used for model evaluation, another CV must be performed in the training data for parameter tuning and preprocessing.

To promote comparison with the evaluation setting presented in the precursor work by Luminari et al. [14], two evaluation settings are considered. The first setting (**setting I**) purposefully corresponds to an approach similar to the one followed by Luminari et al. [14], where the preprocessed data using the two configurations of feature selection (Methods section) are subjected to internal bootstrapping for evaluation purposes. The second setting (**setting II**) guarantees the soundness of the acquired predictability levels by ensuring that feature selection step is performed inside each fold of the nested cross-validation, as previously described. Direct comparison between the two settings should not be attempted given the diversity of the underlying evaluation principles.

A core contribution of this study is the possibility to create a reliable decision support system that can help decide the intensity of the treatment a patient must undergo, with a positive prediction indicating that a more aggressive regime is necessary. Attending to this observation, the following evaluation metrics were chosen: (i) AUC as an overall indicator of how the predictor performs when the decision threshold is not optimized; (ii) recall and precision to ensure that the predictor does not skew towards the majority class, especially important due to the imbalanced data nature; and (iii) specificity to guarantee the identification of patients who will react well to the standard treatment, avoiding the prescription of an unnecessarily stronger chemotherapy regimen. Accuracy, although presenting an useful overall indication of the predictor’s performance, is not considered here due to the imbalanced nature of the data. The metric optimized in the hyperparameterization step is the F1 score, so that the classifiers can attain a balanced performance in both precision and recall. Precision-Recall curves, together with explainability models, are further provided to offer a more comprehensive comparison of the predictors.

### Predictive performance under state-of-the-art ML

The initial phase of feature selection by the Mutual Information and Wilcoxon Rank Sum algorithms identified a total of 250 features out of the initial 770. These features correspond to 248 genes and the clinical variables *stage* and $\textit{LMR}{>} \textit{2.1}$. The work of Luminari et al. [14] found, during a first analysis, a 13-gene signature positively correlated with iPET2 in addition to the variable $\textit{LMR}{>} \textit{2.1}$. In comparison, we find that our most influential feature set contains 9 out of the 13 genes and the $\textit{LMR}{>} \textit{2.1}$ variable. The selection of the variable *stage* by our algorithm points to a relation with the target iPET2 not identified through the multivariate logistic analysis performed by Luminari et al. [14]. The second and final phase of feature selection, performed by the algorithm SVM-RFE, identified a set of 14 genes. Out of these 14 genes, only 2 were also found in the 13-gene signature identified by Luminari et al. [14], indicating a discrepancy between the two gene sets and confirming the difficulty of the task of identifying a concise set of discriminative genes.

In order to provide a reference random baseline for the interpretation of classification results, we performed twenty iterations of training and validation of a random classifier in our dataset. It is the convergence value of these metrics that should be interpreted as the reference minimum value for this classification task, namely, a precision of 0.21, and a recall, specificity and AUC of approximately 0.5.

**Fig. 3:**
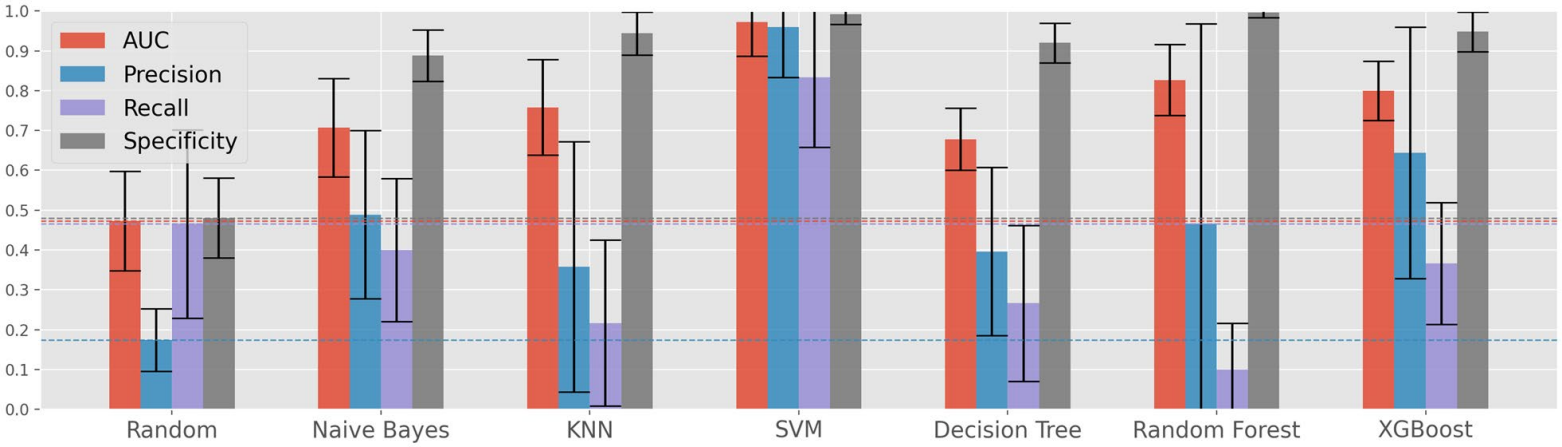
Optimized classifier’s performance in the prediction of iPET2 (setting I)

Results for setting I are presented in Fig. 3, where each color encodes a given performance metric and the horizontal lines correspond to the random classifier’s results in said metric. We can observe that the combination of our two feature selections steps, data balancing using SVM-SMOTE and the classifier SVM, can achieve a superior mean result of 0.97 AUC (against an AUC of 0.84 obtained by Luminari et al. [14]). The high predictive power of an SVM in this setting is to be expected due to its recurrent good performance in this type of data [53, 54], and the fact that is being paired with the SVM-SMOTE data balancing technique and the SVM-RFE feature selection algorithm, both using an SVM as the base of its decisions.

**Fig. 4:**
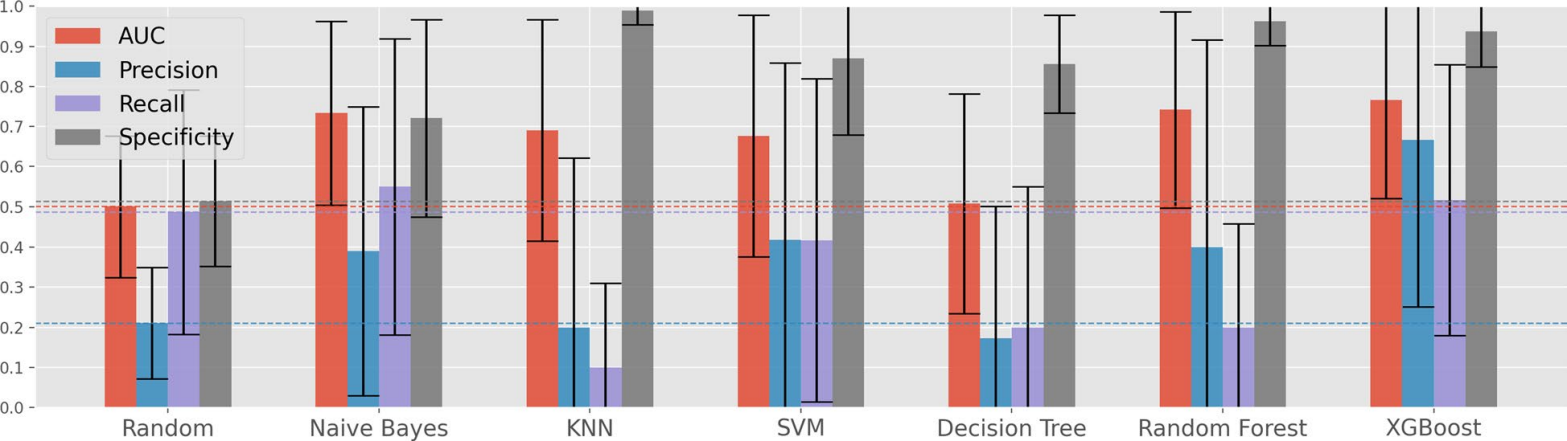
Optimized classifier’s performance in the prediction of iPET2 (setting II)

The results obtained under setting II are presented in Fig. 4. In this case, the XGBoost algorithm achieves significantly better results than the SVM, the previous best performer, with an AUC of 0.77, a precision of 0.67, a recall of 0.52 and a specificity of 0.94.

In contrast, KNN has notably poor performance in comparison with the defined baseline, which can be explained by two major factors: (i) a high percentage of outliers in our dataset; and (ii) high gene expression variability, leading to inflated differences between individuals that belong to the same class. Two out of the three associative classifiers, Decision Tree (DT) and Random Forest (RF), also present comparable performance to the random classifier, suggesting that a decision tree is unable to fully take advantage of all the discriminative gene interactions present in the dataset. Since the RF and XGBoost classifiers are both ensembles of DTs, the discrepancy between their results must originate due to the embed feature engineering capabilities of XGBoost and differences on the pursued bagging and boosting strategies. Since bagging uses multiple independently trained DTs to make predictions, the fact that a DT cannot assimilate the knowledge in the data will lead to an overall lack in performance for the RF. Boosting on the other hand trains the trees iteratively, allowing for each consequent model to improve where the previous one failed, resulting in a classifier that can better model more complex interactions.

Overall, it is observable that all the classifiers can attain a high specificity, but at the cost of a reduced precision and recall. In other words, classifiers have an easier time correctly classifying patients with negative iPET2, guaranteeing that the patients that will react well to the ABVD regimen are correctly identified. The main difficulty with this predictive problem is in the correct classification of positive patients, possibly due to the low number of samples of this class. In addition, treatment response is being assessed with regards to iPET2 results, which may not be an optimal representative of the true quality of patient response to ABVD chemotherapy.

**Fig. 5:**
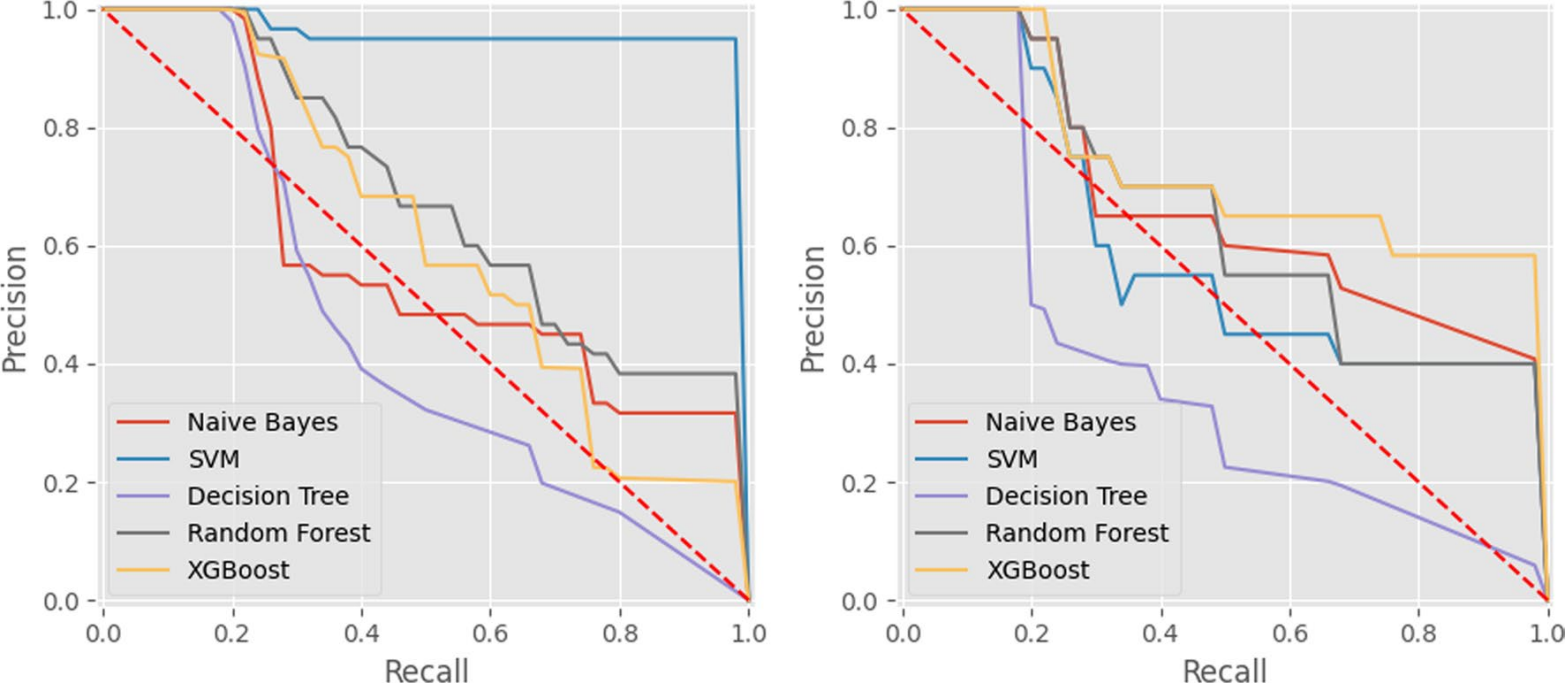
Precision-Recall curves for configuration one (left) and configuration two (right) for each machine learning model

The predictive results can be further analyzed through the study of the classifiers’ Precision-Recall curves presented in Fig. 5. KNN is omitted from these graphics due to its dependence on an ineffective method to calculate the appropriate thresholds.

**Fig. 6:**
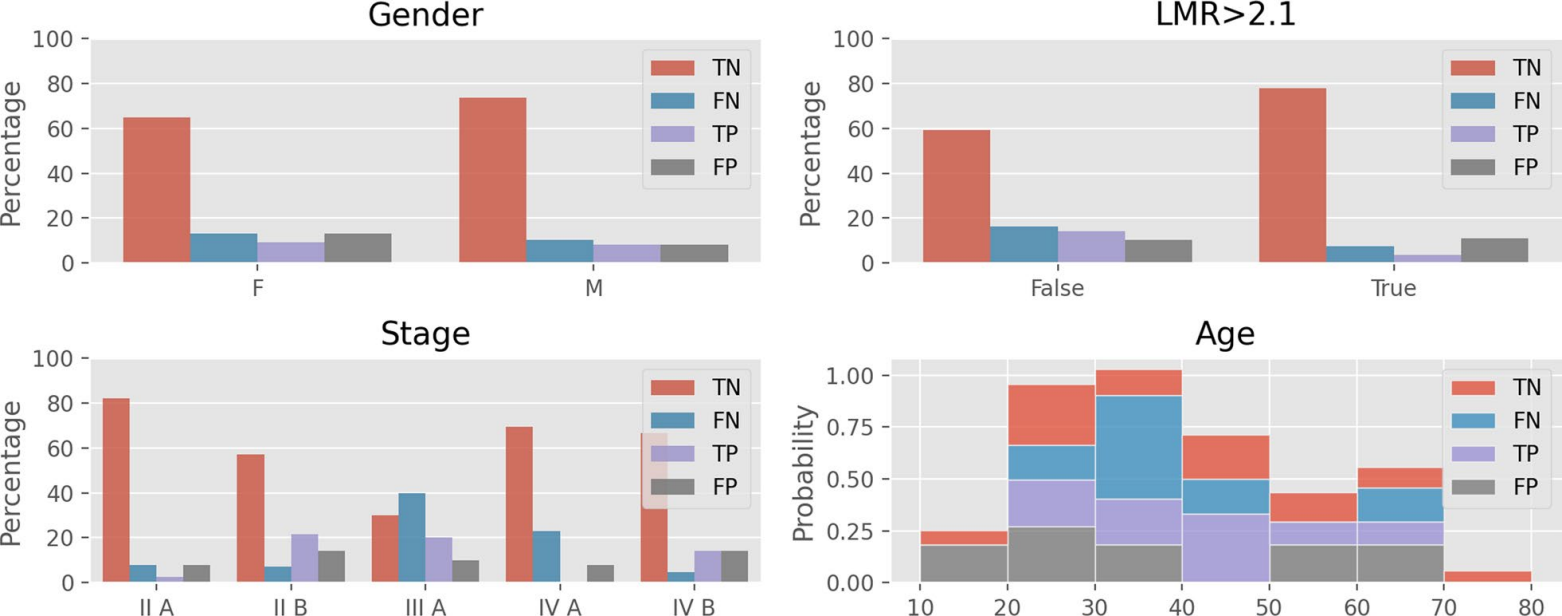
Distribution of predictions for the clinical variables (gender, LMR, stage of the disease and age)

It is in our interest to better understand what leads a certain patient to be misclassified by our models. We plot some of the characteristics of the correctly classified individuals against wrongly classified ones by the best predictor in the second configuration, XGBoost. This analysis is provided for the four clinical variables, *age*, *gender*, *stage* and $\textit{LMR}{>} \textit{2.1}$ in Fig. 6. Starting with the variable *gender*, no significant trend is noted. $\textit{LMR}{>} \textit{2.1}$ on the other hand shows a more clear inclination for correctly classifying positive cases when this variable is “False”, with the percentage of True Positives (TP) being substantially higher than the False Positives (FP) percentage. Regarding the *stage* variable, the values “I A” and “III B” are omitted due to the low number of samples corresponding to each one. In the observation of the remaining values only “III A” shows a significant deviation from the others, with all the positive cases correctly predicted but with a low performance in the false cases. The final plot is dedicated to the variable *age* and is presented in a stacked view, where the bins encompassing a 10-year period from each class (TN, FN, TP or FP) are stacked to facilitate a comparative analysis between them. We can then recognize that the majority of False Negatives (FN) occur in patients between 30 and 40 years, and the False Positives (FP) are more evenly distributed with a slightly higher concentration in patients between 20 to 30 years.

**Fig. 7:**
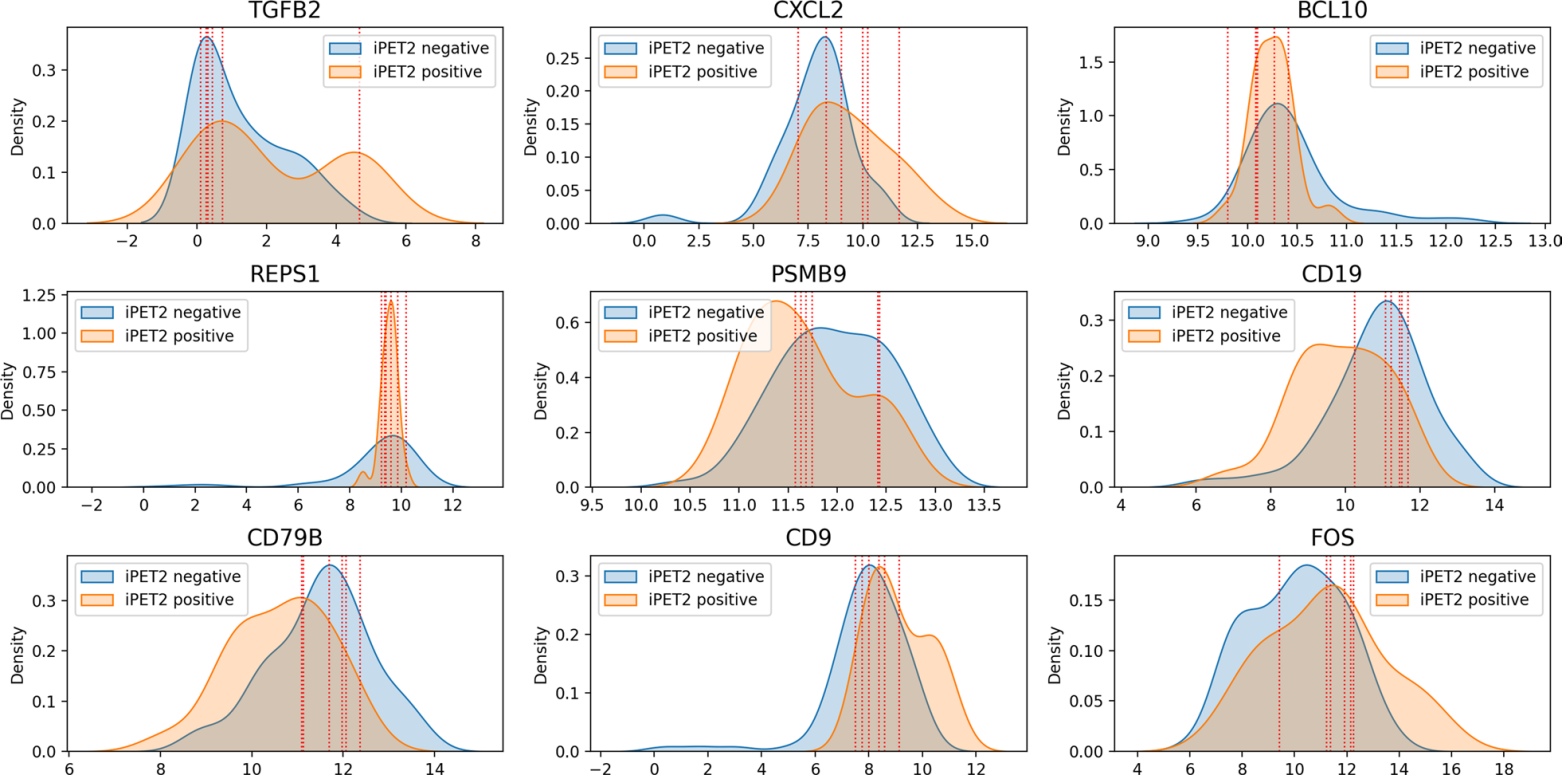
Distribution of top discriminative genes according to Mutual Information with highlighted values (red vertical lines) corresponding to false positive and negative predictions

As some of our results indicate, the majority of useful information about how the patient will react to treatment is contained on gene expression features, and therefore it is imperative that the analysis of the factors inducing wrong classifications be extended towards these features. In Fig. 7 we can see the distributions of the nine more discriminative genes according to their Mutual Information [31] with the response outcome. The red vertical lines highlight the expression levels associated with the six patients that were misclassified by all of the assessed predictive models.

We can then observe that, as expected, the majority of the highlighted values are found in the intersection of both distributions, where the classification is harder to perform.

### Bicluster-based Space transformation

The results presented until here correspond to the classification task in a feature space reduced by a composition of feature selection procedures. This approach lacks the ability to effectively represent the complex gene interactions responsible for the outcome of the patient. In accordance with the introduced methodology, we capture these interactions through discriminative biclusters using BicPAMS [49]. The found biclusters are then used to create new features representing discriminative and statistically significant gene expression patterns.

**Fig. 8:**
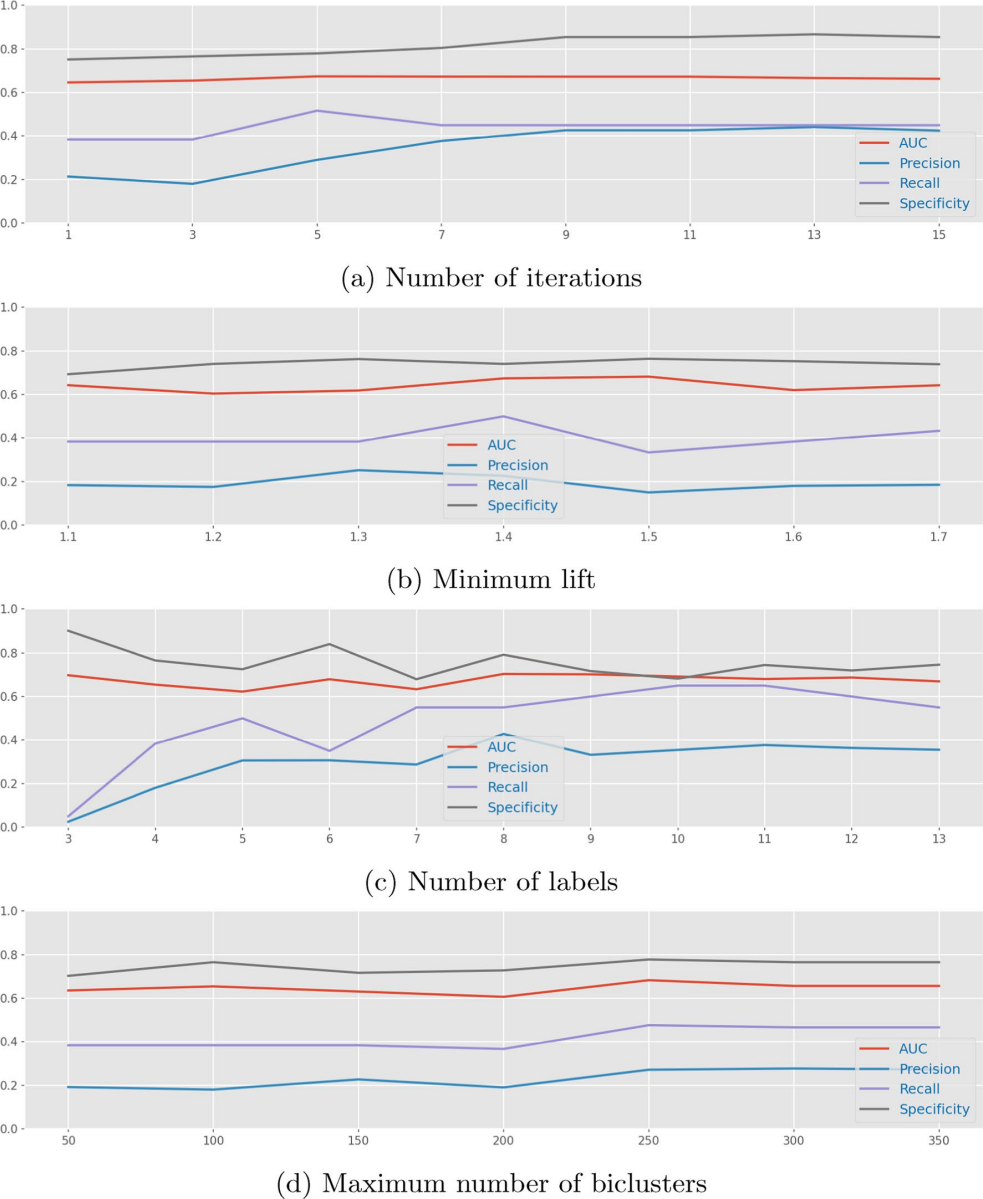
Variation of performance according to multiple parameters

With the goal of better understanding the impact that BicPAMS’ parameters have on predictive performance, we performed a comparative analysis of how distinct parameterizations affect the behavior of a baseline Naive Bayes predictor. The evaluated parameters are: (i) *number of iterations*, indicating how many times the mining process is repeated, masking the found biclusters in each new iteration and forcing the mining process to find other less trivial biclusters but resulting in greater computational cost; (ii) *minimum lift*, a placed threshold to determine whether a given bicluster is sufficiently discriminative [51]; (iii) *number of labels*, corresponding to the number of overlapping gene expression levels [48]; and (iv) *maximum number of biclusters*, the number of mapped features in the transformed data space by the postprocessing filtering of the bottom discriminative biclusters according to their lift. The results for each of these parameters are shown in Fig. 8. We can see in the plotted results that in respect to most of the parameters there is a gain in performance by increasing its respective values, but only until a certain threshold is reached, from which there are no further advantages. The minimum lift is an exception to this, showing almost no effect in this specific problem and pointing to the importance of less discriminative patterns in the learning process.

**Fig. 9:**
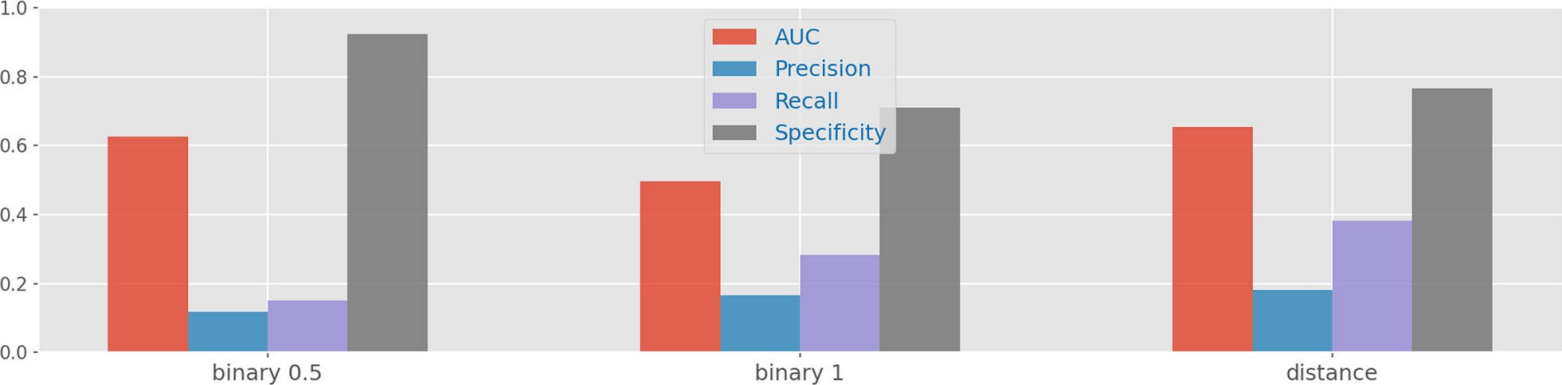
Variation of performance according to the distance criterion

Finally, we further assessed the impact of different dissimilarity functions to assess the how likely is a given gene expression pattern for a specific patient, determining how the values present in the transformed dataset are computed. To this end, we consider both the Euclidean distance and a binary value indicating if a patient possesses a given pattern or not. In the second case, a tolerance threshold can be included to accommodate for noise. Figure 9 shows the performance of a Naive Bayes classifier in three different settings: the binary transformation with thresholds of 0.5 and 1, and the transformation using Euclidean distance. As expected, by using a numeric representation instead of a binary one, there is less loss of information, and consequently, we can achieve better results in the most difficult metrics for this classification task, precision and recall.

**Fig. 10:**
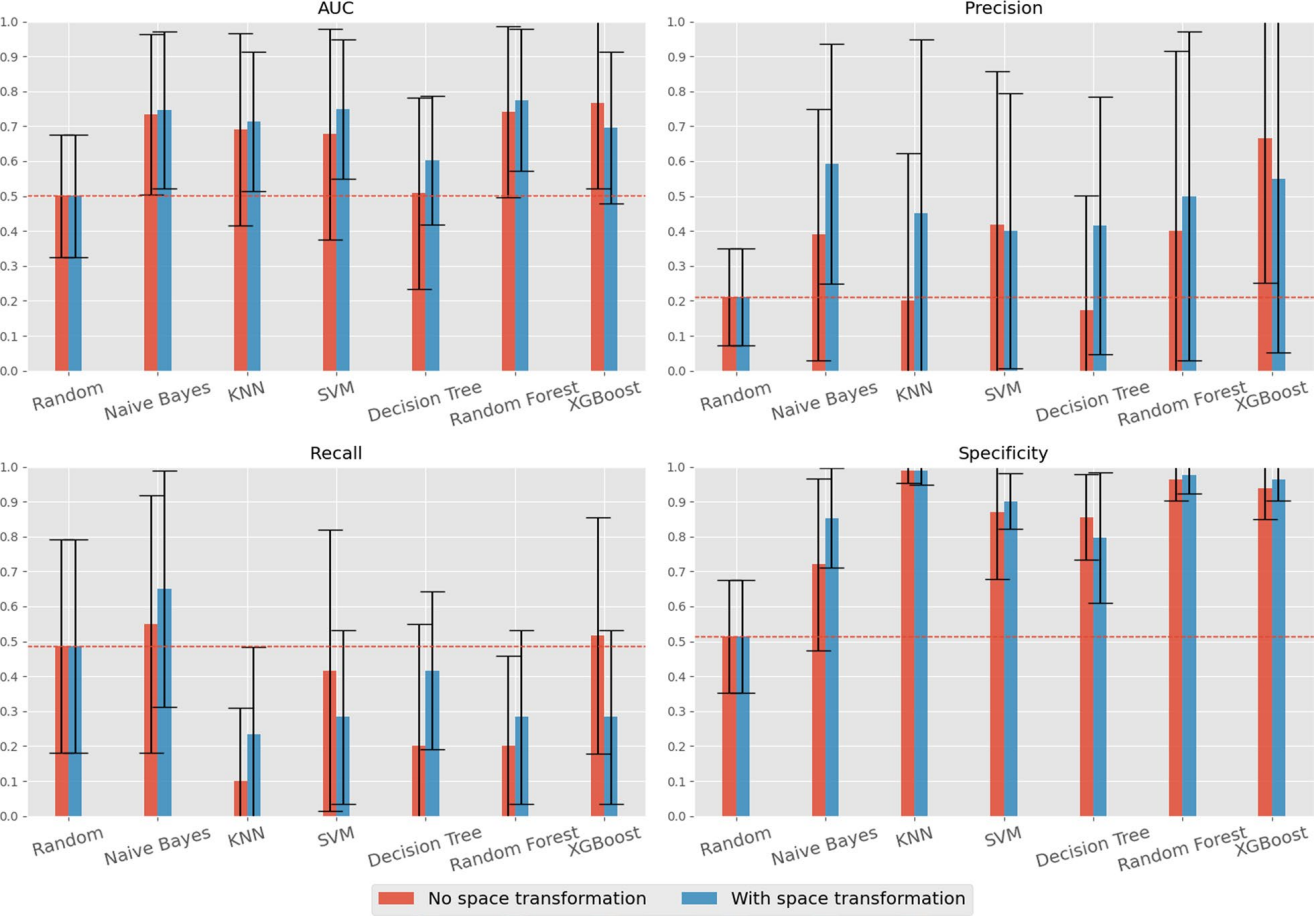
Direct comparison of results with (blue) and without (red) bicluster-based space transformation

To assess the effects of the pattern-centric feature space mapping, we applied the target transformation using BicPAMS algorithm with the following parameters (placed according to previously gathered empirical evidence): number of iterations = 9; minimum lift = 1.3; number of labels = 10; maximum biclusters = 250; and distance criterion = Euclidean distance. Under this configuration, it is possible to carry out a comprehensive exploration of the discriminative biclusters by performing multiple iterations while maintaining a relatively low minimum lift so that the computational requirements do not get too high. The high number of labels guarantees that the found patterns discriminate fine levels of expression while the relatively high number of biclusters ensures that most discriminative and statistically significant biclusters are retrieved. Figure 10 provides a direct comparison of each individual metric between these results and the ones previously obtained by our second setting (Fig. 4).

The gathered results show that the transformed feature space has statistically significant impact on the behavior of the classifiers. SVM and XGBoost, classifiers that already presented good results, are not as significantly affected by this transformation, but all the others benefit from it and present an increase in performance in all the studied metrics.

## Discussion

The ability to better discriminate how a patient will respond to a treatment is essential, especially in the domain of cancer therapy where the majority of treatments are associated with high toxicity and the prognostic exams can be intrusive and expensive. We comprehensively assess the predictability guarantees achieved by state-of-the-art ML models. These models are carefully optimized through the Tree Parzen Estimator algorithm and evaluated in a controlled manner resorting to nested cross-validation. Despite the placed optimization principles, the obtained results still fall short on the predictability power necessary to translate decisions in real-world practice. Transcriptional and iPET2 activity are structurally different, with the former being better positioned to model regulatory responses to treatment, even at the cost of iPET2 discordance.

The high specificity attained by most models (0.94) indicates that the classification models can correctly identify most of the patients that show disease regression after the treatment, but are more susceptible to recognize the positive patients. One possible reason for this bias is the low percentage of positive cases, representing only 20% of the total patients. To correct for this imbalance, we resorted to the use of balancing with SVM-SMOTE and hyperparameterization of the predictive models according to F1 score, an evaluation metric sensitive to the positive samples. Despite these efforts, the best classifier achieved a precision of 0.67 and a recall of 0.52, confirming the impact the difficulty of finding a transcriptional exam resembling the nature of iPET2 activity.

The nature of the target cohort data further introduces generalization challenges to the target prognostication, with low number of samples and high-dimensionality. In addition, the transcriptome profiling is susceptible to the infiltration of non-cancer cells and arbitrarily-high variations to the composition of the target cell population, further contributing to generalization difficulties. Finally, the biological mechanisms underlying diseases such as HL are immensely complex and dependent of interactions between many genes at multiple omics levels.

In order to better represent the complex interactions between genes that originate biological processes, the proposed space transformation offers an elegant way of shifting the learning from individual genes towards patterns of gene expression. By doing this, we group statistically significant and discriminative sets of genes that partake in regulatory modules correlated with a specific outcome of interest. This creates more straightforward conditions to guide the learning of predictive models. We observed that the efficacy of this transformation is proven by the increase in performance of the majority of classifiers in all the studied metrics.

Despite the yield improvements, the gathered results are indicative of the innate difficulty of the target predictive task, claiming for further contributions in this domain able to translate high-dimensional regulatory profiles into actionable and reliable results.

In order to deal with the challenges presented in our work, we suggest the following directions of research: (i) strengthen this methodology by completing the current regulatory stances with complementary omic layers; (ii) further combine the transcription of non-coding RNAs, recently shown to play an important role in HL [55]; (iii) assess the potential increase in performance by using more specialized classification principles best suited to deal with the inherent overlapping class-conditional distributions of expression per gene (Fig. 7); (iv) place a finer description on the quality of treatment response, translating the classification task into an (ordinal) regression task; and (v) further assess the impact of alternative pattern-based feature space transformations on predictive accuracy, namely by resorting to ensembles of biclusters with different characteristics.

## Conclusion

This work introduced a novel methodology to improve the predictive accuracy of HL treatment response after two courses of ABVD chemotherapy against reference predictive levels [14]. This is achieved through a biclustering-based data space transformation that creates a shift from gene-centric to pattern-centric organization of expression data, combined with a thorough optimization of preprocessing procedures and state-of-the-art ML models. The methodology presents increased performance with the proposed space transformation, yielding improvements as large as 20pp in precision and recall, as is the case with Decision Trees. Furthermore, through the application of an ensemble of feature selection procedures, we identify a set of 14 genes highly representative of the result of an FDG-PET after two courses of ABVD chemotherapy. This set only overlaps with the one identified by Luminari et al. [14] in 2 genes, indicating that it can offer a complementary understanding of the HL response to chemotherapy.

## Data Availability

The datasets generated and/or analysed during the current study are available in the National Center for Biotechnology Information Gene Expression Omnibus repository, https://www.ncbi.nlm.nih.gov/geo/query/acc.cgi?acc=GSE132348

## Abbreviations

HL: Hodgkin’s Lymphoma
ABVD: Adriamycin, Bleomycin, Vinblastine and Dacarbazine
ML: Machine learning
FDG-PET: Fuorodeoxyglucose Positron Emission Tomography
IPS: International Prognostic Score
FFPE: Formalin fixed paraffin embedded
FDR: False discovery rate
FC: Fold-change
LMR: Lymphocyte-to-monocyte ratio
SVM-SMOTE: Support vector machine synthetic minority oversampling technique
SVM-RFE: Support vector machine–recursive feature elimination
MI: Mutual information
SVM: Support vector machine
KNN: K-nearest neighbors
BicPAMS: Biclustering based on PAttern Mining Software
CV: Cross-validation
DT: Decision tree
RF: Random forest
TP: True positives
FP: False positives
FN: False negatives
FP: False positives

## Footnotes

1 https://www.ncbi.nlm.nih.gov/geo/query/acc.cgi?acc=GSE125651 (accessed 3/2022)https://www.ncbi.nlm.nih.gov/geo/query/acc.cgi?acc=GSE66417 (accessed 3/2022)https://www.ncbi.nlm.nih.gov/geo/query/acc.cgi?acc=GSE110348 (accessed 3/2022)https://www-ncbi-nlm-nih-gov.ezproxy.u-pec.fr/geo/query/acc.cgi?acc=GSE64234 (accessed 3/2022).

2 https://github.com/Andrempp/challenges_HL

3 https://www.ncbi.nlm.nih.gov/geo/query/acc.cgi?acc=GSE132348 (accessed June 28, 2021).

4 implemented using imbalanced-learn [22]

5 implemented using SciPy [28]

6 implemented using scikit-learn [30]
